# The Role of Gut Microbiota on Idiopathic Pulmonary Fibrosis Mediated by Circulating Inflammatory Proteins: A Two‐Step, Two‐Sample Mendelian Randomization Study

**DOI:** 10.1111/crj.70120

**Published:** 2025-09-09

**Authors:** Hongyu Zhu, Caihua Chen, Haixie Guo, Bo Zhang, Quanteng Hu

**Affiliations:** ^1^ Department of Thoracic Surgery Taizhou Hospital Taizhou Zhejiang China

**Keywords:** circulating inflammatory proteins, gut microbiota, idiopathic pulmonary fibrosis, Mendelian randomization

## Abstract

**Background:**

Persistent inflammation is a crucial characteristic of idiopathic pulmonary fibrosis (IPF). Gut microbiota (GM) contribute to the occurrence and development of several pulmonary diseases through the “gut–lung axis.” The genetic role of GM in IPF and the mediating effect of circulating inflammatory proteins.

**Methods:**

A single nucleotide polymorphism (SNP) was used as an instrumental variable (IV) for exposure to evaluate the causal relationship between exposure and outcome. A two‐step, two‐sample Mendelian randomization study mainly based on an “inverse variance weighted (IVW)” approach was performed to explore the causal relationship between GM and IPF mediated by circulating inflammatory proteins.

**Results:**

The IVW way illustrated 12 taxa (Bacillales, Gastranaerophilales, Selenomonadales, Family XIII, Bacteroidaceae, *Bacteroides*, and *Actinomyces*, *Bifidobacterium*, *Oscillibacter*, *Ruminococcus gnavus* , *Subdoligranulum*, *Veillonella*) of GM and 8 circulating inflammatory proteins (CCL11, CXCL6, CXCL9, CCL8, CCL7, NRTN, STAMPB, and TGFa) had suggestive evidence of causality on IPF. The mediation MR demonstrated the causal pathway from *Actinomyces* to IPF was partly mediated by CCL11 (the mediation effect: 0.063, 95% CI [1.016–1.126]; *p* = 0.004) with a mediation proportion of 13.035%.

**Conclusions:**

These findings may suggest a genetically predicted association between GM and IPF mediated by circulating inflammatory proteins.

## Graphical abstract

We evaluated the causal relationship between gut microbiota and idiopathic pulmonary fibrosis. We evaluated the causal relationship between 91 circulating inflammatory proteins and idiopathic pulmonary fibrosis. The mediation MR demonstrated the causal pathway from *Actinomyces* to IPF was partly mediated by CCL11.

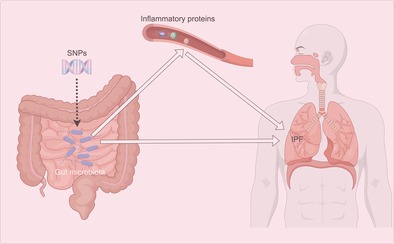

**Abbreviations**
CCL11: C–C motif chemokine ligand 11
CI: confidence interval
COPD: chronic obstructive pulmonary disease
GM: gut microbiota
GWAS: genome‐wide association study
IPF: idiopathic pulmonary fibrosis
IV: instrumental variable
IVW: inverse variance weighted
LD: linkage disequilibrium
MR: Mendelian randomization
MR‐PRESSO: MR‐Egger intercept test and outlier method
OR: odds ratio
SNP: single nucleotide polymorphism

## 1 Introduction

Idiopathic pulmonary fibrosis (IPF), a chronic progressive and incurable interstitial lung disease, is characterized by irreversible fibrotic destruction in the lung and irreversible decline in lung function and organ failure [1, 2]. The most common symptoms of IPF include cough and dyspnea, which would significantly decrease the quality of life [3]. Although epidemic data for IPF is rare, especially in low‐ and middle‐income countries, there are still some data indicating more than 8 and 28 cases per 100 000 people were diagnosed with IPF each year [4, 5]. In addition, the mortality of IPF is high, with the 5‐year survival after diagnosis less than 30% and the median survival limited to 2–3 years without treatment, which places a tremendous burden on society and personal life across the globe [4, 5]. Some risk factors like age, male sex, a positive history of cigarette smoking, and air pollution have been linked to the development of IPF [6]. However, the specific pathogenesis and pathogenic factors still need further exploration, which may contribute to enriching the treatment methods and improving the prognosis of IPF.

In recent years, a novel theory, “gut–lung axis,” has been proposed, indicating the long‐distance cross‐talk between lung and gut [7]. The gut microbiota (GM) is the core part of this theory. Moreover, much evidence has demonstrated that the imbalance of GM may be in connection with lung homeostasis and susceptibility to lung diseases [8]. For example, a study has proved that the dysfunction of GM in chronic obstructive pulmonary disease (COPD) patients was highly related to airway inflammation level, pulmonary function, and the progression of COPD [9]. In addition, a research showed that cannabidiol, a non‐psychoactive derivative of the cannabis plant, could exert an antifibrotic effect via reversing GM imbalance [10]. GM was associated with various lung diseases, but its role in IPF was still unknown.

A vital function of GM is to regulate immune and inflammatory levels [8]. Moreover, emerging shreds of evidence supported that abnormal immune and inflammatory cell levels contribute to the development of IPF due to their secretion of a large number of cytokines, chemokines, and mediators, which could be divided into two types: (1) proinflammatory, such as IL‐6, IL‐1, IL‐13, and TNF‐α, and (2) anti‐inflammatory, such as TGF‐β and IL‐10 [11, 12]. The proinflammatory factors activate fibroblasts and myofibroblasts and induce further fibrotic tissue remodeling to maintain fiber formation and attract immune cells to promote more inflammatory factors and differentiate fibroblasts into myofibroblasts, a positive feedback loop supporting fiber formation [13]. In the study, we aimed to determine the genetic effects of GM on IPF and whether it can be mediated by circulating inflammatory proteins.

## 2 Materials and Methods

### 2.1 Study Design

**FIGURE 1:**
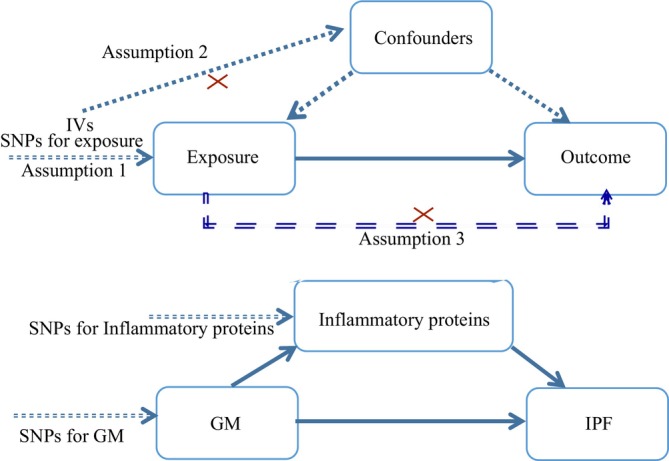
Overview of the MR study design. Three basic assumptions should be followed in MR analysis. (1) Genetic variation has solid association with exposure; (2) Genetic variation should be independent of potential confounders. (3) These genetic tools should only affect outcome via exposure. IPF, idiopathic pulmonary fibrosis; IV, instrumental variable; MR, Mendelian randomization; SNP, single nucleotide polymorphism.

A two‐step Mendelian randomization (MR) study was performed to analyze the causal role of GM on IPF mediated by circulating inflammatory proteins. Firstly, a two‐sample MR was conducted to investigate the association of genetically predicted risk of IPF with GM and circulating inflammatory proteins. Then, the genetic effects of GM on circulating inflammatory proteins (genetically determined mediators) will be explored, and the mediation proportion will be calculated. Three basic assumptions should be followed in MR analysis: (1) Genetic variation has a solid association with exposure; (2) genetic variation should be independent of potential confounders; (3) these genetic tools should only affect outcomes via exposure. The overall design was shown in Figure 1.

### 2.2 Data Source

Single nucleotide polymorphism (SNP) was used as an instrumental variable (IV) for exposure to evaluate the causal relationship between exposure and outcome. All summary statistics were obtained from the GWAS Catalog (genome‐wide association studies; https://gwas.mrcieu.ac.uk/). The GWAS IDs for GM at five levels (phylum, class, order, family, genus) were from Ebi‐a‐GCST90016908 to Ebi‐a‐GCST90017118, including 14 306 samples [14]. The GWAS ID for IPF was Ebi‐a‐GCST90018120, including 437 235 control cases [15]. The accession numbers for circulating inflammatory proteins were from GCST90274758‐GCST90274848, including 14 735 cases [16].

### 2.3 Genetic IV Selection

According to the previous research [17], the parameter for identifying IVs of GM was set as *p* < 1e × 10^−5^. A more stringent cutoff value (*p* < 5e × 10^−6^) was used to select IVs of 91 circulating inflammatory proteins. The threshold for 91 circulating inflammatory proteins was stricter (*p* < 5e × 10^−8^). To reduce the bias caused by linkage disequilibrium (LD), we performed an LD clustering algorithm. IVs were independent as clumped using LD *R* ^2^ < 0.001 within ±10 000 kb distance in 1000 Genomes Project as reference pane. Then, we calculated the *F* statistics of SNPs with this formula: β divided by the square of the standard error. Finally, SNPs with *F* > 10 would be selected and searched in PhenoScanner V2 (http://www.phenoscanner.medschl.cam.ac.uk/) [18]. SNPs violating the second and third assumptions and having potential confounders and bypassing (e.g., age, sex, smoking, and other disease) would be removed. 2082, 1748, and 6 SNPs were selected as the IVs for GM, 91 circulating inflammatory proteins, and IPF, respectively (Table S1).

### 2.4 Statistical Analysis

All statistical analyses were performed using R 4.3.1 (https://www.r‐project.org). The packages: “TwoSampleMR,” “ieugwasr,” and “VariantAnnotation” were applied to execute the two‐sample MR study with five modes: “Weighted median,” “MR Egger,” “Simple mode,” “Weighted mode,” and “Inverse variance weighted (IVW).” The IVW approach was the most precise and robust way and served as the primary mean to estimate the causal effect of exposure on outcome with *p* < 0.05 as the cutoff value. The degree of heterogeneity was tested using Cochran's *Q* statistic. If the null heterogeneity were rejected, the random effects would be instead of the fixed‐effect model. The MR pleiotropy residual sum and outlier method (MR‐PRESSO) combined with the MR‐Egger intercept test were applied to check the evidence of horizontal pleiotropy using the R package “MR‐PRESSO” [19, 20]. The MR‐PRESSO approach would adjust for horizontal pleiotropy by eliminating outliers and comparing the significance of the differences between the original and corrected data. The leave‐one‐out analysis was also employed to determine if a single SNP drove or influenced the MR analysis. The “product of coefficients” method was used to determine how circulating inflammatory proteins‐mediated GM indirectly affect the risk of IPF. β (impact of GM on circulating inflammatory proteins) * β (effect of circulating inflammatory proteins on IPF) is the indirect effect (β). The overall effect (β) = β (impact of GM on IPF). The mediated fraction is the indirect effect (β)/the overall effect (β). Using the delta approach, standard errors for the indirect effects were calculated.

## 3 Results

### 3.1 Total Effect of GM on IPF

**FIGURE 2:**
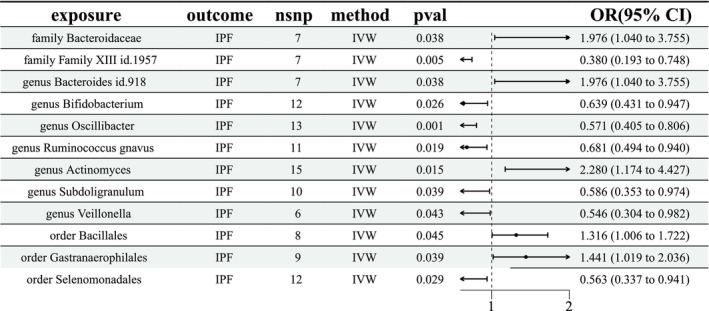
Main results (IVW method) of the causal effect of GM on IPF. CI, confidence interval; GM, gut microbiota; IPF, idiopathic pulmonary fibrosis; IV, instrumental variable; IVW, inverse variance weighted; OR, odds ratio; SNP, single nucleotide polymorphism.

The results of IVW analyses revealed that at the order level, Bacillales (odds ratio (OR) = 1.316, 95% confidence interval (CI) [1.006, 1.722], *p* = 0.045), Gastranaerophilales (OR = 1.441, 95% CI [1.019, 2.036], *p* = 0.039), and Selenomonadales (OR = 0.536, 95% CI [0.337, 0.941], *p* = 0.029) had robust causal associations with the risk of IPF; at the family level, Bacteroidaceae (OR = 1.976, 95% CI [1.040, 3.755], *p* = 0.038) and Family XIII (OR = 0.380, 95% CI [0.193, 0.748], *p* = 0.005) had; at the genus level, *Bacteroides* (OR = 1.976 95% CI [1.040, 3.755], *p* = 0.038), *Bifidobacterium* (OR = 0.639, 95% CI [0.431, 0.947], *p* = 0.026), *Oscillibacter* (OR = 0.571, 95% CI [0.405, 0.806], *p* = 0.001), *Ruminococcus gnavus* (OR = 0.681, 95% CI [0.494, 0.940], *p* = 0.019), *Actinomyces* (OR = 2.280, 95% CI [1.174, 4.427], *p* = 0.015), *Subdoligranulum* (OR = 0.586, 95% CI [0.353, 0.974], *p* = 0.039), and *Veillonella* (OR = 0.546, 95% CI [0.304, 0.982], *p* = 0.043) (Figure 2). No significant evidence of heterogeneity across these results was found using Cochrane *Q* statistics (Table S2). In addition, no definitive horizontal pleiotropy was detected with MR‐PRESSO and MR‐Egger intercept tests (Tables S3 and S4). In the leave‐one‐out sensitivity analysis, no single SNP seriously violated the overall effect of GM on IPF.

### 3.2 Effect of Circulating Inflammatory Proteins on IPF

**FIGURE 3:**
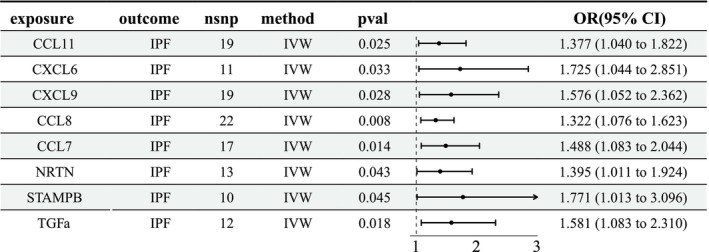
Main results (IVW method) of the causal effect of circulating inflammatory proteins on IPF. CI, confidence interval; IPF, idiopathic pulmonary fibrosis; IV, instrumental variable; IVW, inverse variance weighted; OR, odds ratio; SNP, single nucleotide polymorphism.

The MR analysis using the IVW method demonstrated suggestive evidence of causality of 8 circulating inflammatory proteins on IPF (Figure 3): CCL11 (OR = 1.377, 95% CI [1.040, 1.822], *p* = 0.025), CXCL6 (OR = 1.725, 95% CI [1.044, 2.851], *p* = 0.033), CXCL9 (OR = 1.576, 95% CI [1.052, 2.362], *p* = 0.028), CCL8 (OR = 1.322, 95% CI [1.076, 1.623], *p* = 0.008), CCL7 (OR = 1.488, 95% CI [1.083, 2.044], *p* = 0.014), NRTN (OR = 1.395, 95% CI [1.011, 1.924], *p* = 0.043), STAMPB (OR = 1.771, 95% CI [1.013, 3.096], *p* = 0.045), TGFa (OR = 1.581, 95% CI [1.083, 2.310], *p* = 0.018). No apparent heterogeneity and horizontal pleiotropy were observed (Tables [Link], [Link]–S4). In addition, the leave‐one‐out analysis revealed a single SNP did not drive the overall estimates.

### 3.3 Effect of GM on Circulating Inflammatory Proteins

**FIGURE 4:**
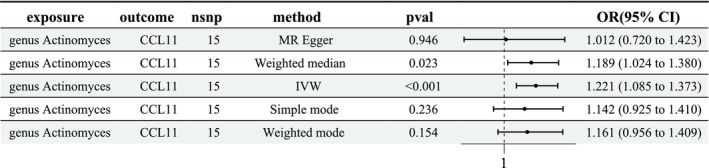
The MR analysis of the causal effect of *Actinomyces* on CCL11. CI, confidence interval; IV, instrumental variable; IVW, inverse variance weighted; OR, odds ratio; SNP, single nucleotide polymorphism.

Next, we investigated the genetic relation between 12 GM and 8 circulating inflammatory proteins. The results showed only the causal role of *Actinomyces* on CCL11 was significant (OR = 1.221, 95% CI [1.085, 1.373], *p* < 0.001; Figure 4). The *p* values derived from Cochran's *Q* statistic were 0.305 and 0.280, hinting no heterogeneity was determined (Table S2). Moreover, the MR‐Egger intercepts test and MR‐PRESSO confirmed no directional horizontal pleiotropy between exposure and outcome (Tables S3 and S4). The leave‐one‐out analysis showed that no particular SNPs substantially impacted the MR analysis.

### 3.4 The Mediation Effect of GM on IPF

Previously, we identified the causal effect of *Actinomyces* on IPF and CCL11 as well as the effect of CCL11 on IPF, suggesting potential mediation of *Actinomyces* to IPF causality via CCL11. Thus, we performed a mediation analysis. In the causal pathway from *Actinomyces* to IPF, the mediation effect of CCL11 was estimated at 0.063 (95% CI [1.016–1.126]; *p* = 0.004) with a mediation proportion of 13.035% (Table 1).

**TABLE 1:** Mediation effect of GM on IPF via inflammatory protein.

Mediator	Total effect	Direct effect A	Direct effect B	Mediation effect	*p*	Mediated Proportion (%)
	β (95% CI)	β (95% CI)	β (95% CI)	β (95% CI)		
CCL11	0.487 (1.174–4.427)	0.199 (1.085–1.373)	0.319 (1.040–1.822)	0.063 (1.016–1.126)	0.004	13.035

*Note:* Total effect: The causal role of gut microbiota on IPF. Direct effect A: The causal role of GM on inflammatory protein. Direct effect B: The causal role of inflammatory protein on IPF. β (indirect effect) = β (direct effect A) * β (direct effect B). The mediated proportion = β (indirect effect)/β (total effect).

Abbreviations: CI, confidence interval; GM, gut microbiota; IPF, idiopathic pulmonary fibrosis.

### 3.5 A Reverse MR Analysis

**FIGURE 5:**
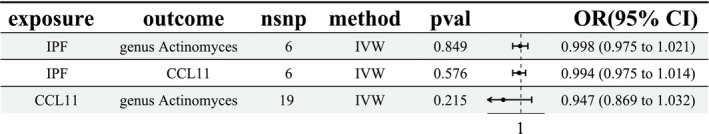
A reverse MR analysis (IVW method) showed the causal effect of IPF on *Actinomyces* and CCL11 as well as CCL11 on *Actinomyces*. CI, confidence interval; GM, gut microbiota; IPF, idiopathic pulmonary fibrosis; IV, instrumental variable; IVW, inverse variance weighted; OR, odds ratio; SNP, single nucleotide polymorphism.

Finally, we performed a reverse MR analysis. The results demonstrated weak evidence of a potential causal effect of IPF on *Actinomyces* (OR = 0.998, 95% CI [0.975, 1.021], *p* = 0.849) and CCL11 (OR = 0.994, 95% CI [0.975, 1.014], *p* = 0.576) (Figure 5). Similar results were observed in the genetic role of CCL11 in *Actinomyces* (OR = 0.947, 95% CI [0.869, 1.032], *p* = 0.215). Furthermore, no evidence for heterogeneity and horizontal pleiotropy was observed (Tables [Link], [Link]–S4).

## 4 Discussion

In the present study, we executed a two‐sample MR analysis to seek the causal relationship between GM and IPF. In the past decade, numerous references have proved that GM contributes to the occurrence and development of several pulmonary diseases, like COPD, through the “gut–lung axis” [8]. The current MR study illustrated the increased proportion of five taxa (Bacillales, Gastranaerophilales, Bacteroidaceae, *Bacteroides*, and *Actinomyces*) would enhance the risk of IPF; whereas, the decreased abundance of seven taxa (Selenomonadales, Family XIII, *Bifidobacterium*, *Oscillibacter*, *R. gnavus* , *Subdoligranulum*, *Veillonella*) would reduce. Regulating immune and inflammatory levels is one of the fundamental biological functions that GM plays. Furthermore, persistent inflammation is a key characteristic of IPF, widely involved in various important pathogenic processes of IPF [11]. The IVW approach showed 8 circulating inflammatory proteins (CCL11, CXCL6, CXCL9, CCL8, CCL7, NRTN, STAMPB, and TGFa) had disadvantageous effects on IPF. The evidence above hinted that GM may increase the development risk of IPF via circulating inflammatory proteins. Hence, we implemented a mediation analysis to validate the hypothesis. Among the 12 taxa of GM, only *Actinomyces* and CCL11 had a significant connection. The proportion of indirect effects of CCL11 was 0.063, accounting for 13.035% of the total effect. Those data indicated that CCL11 was a critical moderator in the causal pathway from *Actinomyces* to IPF.

*Actinomyces* is a genus of bacteria that includes a wide range of species and is found in both human and animal bodies in addition to soil microbial ecosystems [21]. *Actinomyces* belong to Gram‐positive bacilli that are facultatively anaerobic or microaerophilic rods. Actinomycosis is the most prevalent infection linked to actinomycosis [22]. Sulfur particles, or little yellow clumps, can induce persistent granulomatous infections once an actinomycete has infected the host. Previously, a study has shown that the main commensals of gut *Actinomyces* in severe COVID‐19 patients were increased and closely related to the severity of the condition [23]. Furthermore, gut *Actinomyces* could secrete membrane vesicles rich in lipoteichoic acid, which promotes inflammation signaling through TLR2 [24], which exhibited high‐density distribution on the surface of alveolar macrophages in the lungs and could regulate the secretion of proinflammatory cytokines. In addition, *Actinomyces* was found to induce inflammatory cell death, including pyroptosis, with this effect being partially mediated by the TLR2 pathway [25, 26] The abundance of *Actinomyces* increased after gastrectomy. After restoring intestinal homeostasis, early postoperative inflammation was reduced, and immune function was enhanced [27]. Additionally, another literature suggested that increased gut *Actinomyces* may be related to host immunity related to tuberculosis status. The above data indicated that gut *Actinomyces* may provide biological functions for regulating host immunity and inflammatory response [28].

C–C motif chemokine ligand 11 (CCL11), one of several chemokine genes clustered on the q arm of chromosome 17, is a secreted protein superfamily involved in immune regulation and inflammatory processes, with chemotactic activity towards eosinophils [29, 30]. It is believed to be associated with eosinophilic inflammatory diseases such as atopic dermatitis and allergic rhinitis [29]. The expression of CCL11 in IPF was increased approximately twofold and highly related to pulmonary function. Moreover, inhibiting the release of CCL11 from T helper 2 cells could control the transition from an acute inflammatory response to chronic fibrosis [31]. However, there is currently no research reporting the specific role of CCL11 in IPF. CCL11 can promote the binding of Th2‐related cytokines to TLR, promoting the production of immune cells (eosinophils, macrophages, T cells, and B cells) [32]. This process can be inhibited by Th1‐related interferons. CCL11 can also induce fibroblast production, eosinophil infiltration, and Th2 immune response through different pathways [33]. CCL11 can directly enhance the extracellular matrix generation and microscopic proliferation ability of human lung fibroblasts in vitro, and blocking CCL11 signaling could ameliorate fibrosis in mice [34, 35]. In the study, we assessed the role of the pathway from *Actinomyces* to CCL11 on IPF. In future research, utilizing these target antibodies as therapeutic methods for liver fibrosis models will certainly add more translational value to current findings.

In the current study, we explored the causal role of GM on IPF and the mediation effect of circulating inflammatory proteins with a two‐sample, two‐step MR study. MR analysis is a powerful statistical strategy based on whole genome association analysis to infer causal relationships between exposure and outcome in epidemiology, which could simulate randomized controlled trials with a lower cost and less risk of a reverse causal effect. However, there were still some shortcomings: (1) All data were gathered from the public dataset, so we cannot evaluate all of the potential horizontal pleiotropy and heterogeneity. (2) Because the GWAS data used are mainly based on the European population, the generalizability of this conclusion is limited, and it is necessary to validate this conclusion in other populations. (3) We used different threshold criteria (*p* < 1 × 10^−5^ for GM; *p* < 5 × 10^−8^ for inflammatory proteins). Some SNPs that are weakly associated with exposure factors may be influenced by other unknown factors, thereby affecting the reliability of causal effects. (4) The results showed only the pathway: *Actinomyces*‐CCL11‐IPF was significant. Herein, we only analyzed the role of 211 bacteria and 91 circulating inflammatory proteins on IPF, and only 14 306 samples for bacteria and 14 735 cases for circulating inflammatory proteins were collected, which may lead to bias in results. Hence, the study including more GM and inflammatory proteins, as well as larger sample sizes, is necessary. (5) CCL11 partly mediated the pathway from GM to IPF. Nevertheless, other mediators we have yet to study may also exist, requiring further investigation.

## 5 Conclusions

Herein, we concluded that GM and circulating inflammatory proteins had a genetically predicted association with IPF. Of note, the causal effect of *Actinomyces* on IPF was, to a large extent, mediated by CCL11.

## Author Contributions

Quanteng Hu and Bo Zhang designed the study and revised the manuscript. Hongyu Zhu and Caihua Chen analyzed the data, performed the experiment, and wrote the manuscript. Haixie Guo was involved in data collection and statistical analysis and reviewed the manuscript. All authors read and approved the final manuscript.

## Ethics Statement

All procedures performed in studies involving human participants were in accordance with the ethical standards of the institutional and/or national research committee and with the 1964 Helsinki declaration and its later amendments or comparable ethical standards.

## Conflicts of Interest

The authors declare no conflicts of interest.

## Supporting information


**Table S1:** The selection and harmonization of IVs.

**Table S2:** The heterogeneity test using Cochrane Q statistics.

**Table S3:** The horizontal pleiotropy test using MR‐Egger intercept test.

**Table S4:** The horizontal pleiotropy test using MR‐PRESSO.

## Data Availability

The datasets analyzed during the present study were obtained from GWAS Catalog (genome‐wide association studies; https://gwas.mrcieu.ac.uk/). The GWAS ids for GM at five levels (phylum, class, order, family, genus) were from Ebi‐a‐GCST90016908 to Ebi‐a‐GCST90017118. The GWAS ID for IPF was Ebi‐a‐GCST90018120. The accession numbers for circulating inflammatory proteins were from GCST90274758–GCST90274848, including 14 735 cases.
